# Use of Membrane Techniques for Removal and Recovery of Nutrients from Liquid Fraction of Anaerobic Digestate

**DOI:** 10.3390/membranes15020045

**Published:** 2025-02-02

**Authors:** Magdalena Zielińska, Katarzyna Bułkowska

**Affiliations:** Department of Environmental Biotechnology, University of Warmia and Mazury in Olsztyn, Słoneczna St. 45G, 10-709 Olsztyn, Poland; katarzyna.bulkowska@uwm.edu.pl

**Keywords:** anaerobic digestate, nutrient recovery, membrane separation, nitrogen, phosphorus, circular economy, sustainable agriculture

## Abstract

This review focuses on the use of membrane techniques to recover nutrients from the liquid fraction of digestate (LFD) and emphasizes their role in promoting the principles of the circular economy. A range of membrane separation processes are examined, including microfiltration (MF), ultrafiltration (UF), nanofiltration (NF), reverse osmosis (RO), forward osmosis (FO), membrane distillation (MD) and new tools and techniques such as membrane contactors (MCs) with gas-permeable membranes (GPMs) and electrodialysis (ED). Key aspects that are analyzed include the nutrient concentration efficiency, integration with biological processes and strategies to mitigate challenges such as fouling, high energy requirements and scalability. In addition, innovative hybrid systems and pretreatment techniques are examined for their potential to improve the recovery rates and sustainability. The review also addresses the economic and technical barriers to the full-scale application of these technologies and identifies future research directions, such as improving the membrane materials and reducing the energy consumption. The comprehensive assessment of these processes highlights their contribution to sustainable nutrient management and bio-based fertilizer production.

## 1. Introduction

Digestate, the by-product of anaerobic digestion (AD) of organic waste (such as sewage sludge, agricultural waste and other lignocellulosic materials), is increasingly recognized as a secondary resource for the recovery of nutrients, especially nitrogen (N) and phosphorus (P). Its composition, variability and potential for valorization make it a crucial component in the development of circular economy strategies, particularly the production of bio-based fertilizers and water reclamation. The annual production of digestate in the European Union is estimated at 180 million tons, containing 2–5 kg N/m^3^ and 0.5–1.5 kg P/m^3^ [1]. Digestate possesses high nutrient content; the N content is between 1.6% and 21% (dry matter (DM)), while the P content varies between 0.1% and 3.5% DM [2]. Its nutrient profile can vary greatly depending on the fermentation process and source material, with digestate from livestock manure being particularly rich in nutrients. These properties underline the potential of digestate as a sustainable alternative to synthetic fertilizers.

Digestate is increasingly being considered as a feedstock for nutrient recovery, with technologies such as ammonia stripping, struvite precipitation and membrane filtration being used to extract N and P for fertilizer production. Membrane technologies, including pressure-driven processes (microfiltration (MF), ultrafiltration (UF), nanofiltration (NF), reverse osmosis (RO)), forward osmosis (FO), membrane distillation (MD), membrane contactors (MCs) with gas-permeable membranes (GPMs) and electrodialysis (ED), show promise in concentrating nutrients in a transportable and reusable form. These methods simultaneously produce high-quality water and enable the dual benefits of nutrient recovery and water recycling [3]. Despite these advantages, there are significant barriers to practical application, such as membrane fouling, ammonia volatilization and high economic costs. In particular, fouling and clogging increase the maintenance costs and limit the operational efficiency, requiring robust pretreatment and cleaning protocols.

Membrane filtration techniques are often used to maximize nutrient recovery from the liquid fraction of digestate (LFD) [2]. These techniques effectively remove suspended solids and allow the extraction of N and P in a concentrated form, suitable for direct agricultural use or further processing into commercial fertilizers. Pretreatment to reduce suspended solids (SS) is essential for membrane filtration, as high total suspended solids (TSS) content can reduce the membrane efficiency and lead to fouling [4].

The use of LFD as a biofertilizer offers considerable ecological and economic advantages. By replacing mineral fertilizers, it reduces the need for synthetic N, which is a major contributor to greenhouse gas emissions in conventional fertilizer production [5]. The ammonia in LFD can be converted to nitrate when applied to the soil, making the N readily available for plant uptake and reducing the N leaching and emissions associated with synthetic fertilizers [6]. Compared to conventional fertilizer production, nutrient recovery reduces energy consumption and CO_2_ emissions, contributing to sustainable agricultural practices. Integrating digestate management into broader value chains could enable it to serve as a cornerstone of innovative, environmentally friendly fertilizer production systems.

Membrane-based nutrient recovery technologies have the advantage of not requiring the use of as many chemicals as other methods. However, the high capital and operating costs associated with their energy-intensive processes remain a major barrier to their widespread adoption. In addition, the low market price of bio-based fertilizers limits the economic incentives for producers, highlighting the need for policy support and market development [7].

The current research on membrane-based nutrient recovery is largely focused on laboratory-scale studies and offers few examples of pilot- and large-scale applications. Scaling up these efforts to larger scales is essential to demonstrate economic feasibility and optimize operations. The hybridization of membrane technologies with other treatment systems offers the opportunity to improve the efficiency and overcome technical challenges.

The recovery of nutrients from anaerobic digestate has emerged as a crucial area in sustainable waste management and circular economy strategies. Membrane technologies offer solutions for nutrient recovery and water reuse due to their adaptability, efficiency and versatility. While several papers have already addressed membrane processes for wastewater treatment [8] and nutrient recovery [9], this review contributes by integrating the latest developments in membrane technologies. This review stands out from others by addressing innovations in membrane design, such as advanced hybrid systems that combine membrane techniques with biological and chemical processes to improve the efficiency and scalability of nutrient recovery. In contrast to previous works, this review systematically addresses the integration of cutting-edge technologies, including GPMs, MCs and ED, into nutrient recovery. These methods offer unprecedented efficiency in the recovery of ammonia and P while reducing the energy consumption and environmental impact. In addition, this review emphasizes pretreatment, such as fouling-resistant membranes and energy-efficient designs, which are critical to overcoming the operational challenges that have hindered large-scale application in the past.

## 2. Separation of Digestate

Given the high water content of digestate, the costs of transporting, storing and spreading it are considerable, justifying the need for solid–liquid separation [10]. The separation of fermentation residues into solid and liquid fractions forms the basis of most nutrient recovery processes. Separation in this manner not only reduces the volume of the digestate, but also optimizes subsequent treatment options, especially for LFD [2]. Solid–liquid separation produces two distinct phases: a nutrient-rich LFD and a solid fraction that can be used in agriculture or further processed [11]. Separation is crucial in converting the digestate into fractions that will enable efficient nutrient management and recovery [1]. After separation, the LFD typically contains about 85% of the total NH_4_^+^-N and a considerable amount of K, while about 35–45% of the total P remains in this fraction, depending on the separation method used [12]. The high NH_4_^+^-N content makes the LFD comparable to synthetic fertilizers in terms of N availability, so it can be used as a biofertilizer in crop production. In addition, the N/P ratio is often higher in the LFD than in the solid phase [6], creating a balanced nutrient profile that benefits crops while preventing P accumulation in the soil. The typical characteristics of LFD are shown in Table 1.

The efficiency of nutrient separation can be influenced by the type of equipment used. Various separation methods and tools are used for the preparation of AD residues, including centrifugation, belt filters, screw presses, vibrating screens and chemically enhanced methods [11,12,13]. Screw presses, centrifuges and belt filters are usually used as the first step of separation; they separate the fermentation residue into a more concentrated solid part and a liquid fraction.

To improve the removal of solids, chemical conditioning agents such as polyaluminum chloride are commonly used to improve solid separation [14]. This is beneficial for the recovery of nutrients by advanced processes such as membrane filtration. However, these additives must comply with local regulations regarding environmental pollution. The solid fraction, which usually contains between 20 and 25% total solids (TS) by weight, is rich in organic N and P and is therefore suitable as a soil conditioner or as an input for further treatment steps, such as drying or composting, to produce a commercially viable fertilizer product [1]. The liquid fraction, generally characterized by lower DM content of 3 to 7% by weight, contains a high concentration of ammoniacal N, suitable for various recovery and treatment processes [2].

Each method results in a different nutrient distribution, with centrifuges generally achieving higher dry matter and P removal than screw presses, albeit at a higher energy cost [15]. Screw presses and vibrating screens lead to a higher share of total Kjeldahl nitrogen (TKN) in suspended particles out of the total TKN in the LFD (46–65%), while the use of screw presses with coagulants and centrifugation lowers this share to 11–38%, reflecting the better separation of N into the LFD. The application of centrifugation and a screw press with coagulants results in an LFD with lower TS content and is thus more effective in producing a liquid stream suitable for further processing. Conversely, screw presses and vibrating screens used alone can leave similar TS concentrations in the LFD as in the raw digestate, reducing the efficiency of subsequent treatment steps [13].

Due to the presence of complex organic substances, LFD is only biodegradable to a limited extent. This complexity is due to the presence of humic substances and suspended solids, with 60–96% of COD present in particles (>1.2 µm), 2–27% in colloidal form (1.2 µm–1 kDa) and 2–18% in dissolved form (<1 kDa) [13]. High nutrient concentrations, including 1.5–6.5 g total nitrogen (TN)/L and elevated levels of NH_4_^+^-N, K^+^ and phosphate (PO_4_^3−^), add to the treatment challenges and limit the direct application of LFD as a fertilizer due to regulatory restrictions such as the European Nitrates Directive.

Due to the poor biodegradability of LFD, conventional aerobic treatments are inefficient. Its high COD and nutrient concentrations make direct disposal difficult, as land application can lead to environmental problems such as N leaching and groundwater contamination. Recent studies have explored alternative treatment methods, including microalgae cultivation, ammonia recovery and struvite precipitation. However, the high solids content and turbidity of LFD hinder applications such as the cultivation of microalgae, which require light for optimal growth [16]. Therefore, pretreatment to reduce the turbidity is essential for nutrient recovery in these applications.

**Table 1:** Characteristics of LFD.

Characteristic	Unit	[17]		[18]	[19]	[20]	[21]	[22]	[23]	[24]	[25]
Substrate for AD		pig slurry and plant materials		liquid manure and corn silage	pig manure	food waste	livestock manure and agricultural residue	chicken manure	sewage sludge	swine wastewater	cow manure
L/S separation method				mechanical dewatering			filtration				
pH	–	8.03	7.54	NA	8.83	7.10	NA	NA	4.43	8.16	8.77
Total Solids (TS)	%	2.32	1.45	NA	3763.33 mg/L	NA	NA	2.63	2.84 g/kg	NA	8.8
Volatile Solids (VS)	% TS	67.2	66.1	NA	NA	NA	NA	49.13	1.35 g/kg	NA	5.37
TSS	mg/L	NA	NA	10,700	NA	NA	NA	NA	7.33	1041	NA
TOC	mg/L	NA	NA	NA	NA	2140	NA	NA	NA	588.25	36% TS
COD	mg/L	NA	NA	13,400	NA	NA	NA	NA	7413	1009.50	NA
SCOD	mg/L	NA	NA	11,700	NA	NA	NA	NA	6417	NA	NA
NH_4_^+^-N	mg/L	NA	NA	2800	NA	2360	NA	3750	128.67	532.36	4.4% TS
TN	mg/L	9.70% TS	14.8% TS	5800	1536.80	3100	331.33	4500	NA	564.50	8.4% TS
TC-to-TN ratio	–	4.40	2.38	NA	NA	NA	NA	NA	NA	NA	4.2
Nitrate nitrogen	mg/L	NA	NA	NA	NA	66.80	NA	NA	1013.33	NA	TS
VFA	mg/L	NA	NA	NA	NA	NA	NA	56.38	3097	NA	NA
P	mg/L	2.44% TS	1.66% TS	600	17.97	256	153.62	NA	810	41.94	4.3% TS
K	mg/L	5.78% TS	9.24% TS	NA	NA	960	470.25	NA	NA	303.13	10.7% TS
Electrical conductivity (EC)	mS/cm	NA	NA	NA	NA	25.8	NA	NA	NA	5.46	4.6
Calcium	mg/L	NA	NA	NA	NA	NA	12.90	NA	NA	39.41	NA
Sodium	mg/L	NA	NA	NA	NA	1590	106.2	NA	NA	104.80	NA
Magnesium	mg/L	NA	NA	NA	NA	NA	3.38	NA	NA	15.59	3.6%TS
Cadmium	mg/L	NA	NA	NA	NA	NA	0.01	NA	NA	2.31	0.1 mg/kg TS
Chromium	mg/L	NA	NA	NA	NA	NA	0.05	NA	NA	NA	10 mg/kg TS
Iron	mg/L	NA	NA	NA	NA	1.01	4.20	NA	9.33	NA	0.25% TS
Manganese	mg/L	NA	NA	NA	NA	0.04	0.02	NA	NA	NA	360 mg/kg TS
Copper	mg/L	NA	NA	NA	NA	0.33	0.40	NA	7.00	NA	NA
Zinc	mg/L	NA	NA	NA	NA	0.10	2.42	NA	14.00	4.55	135 mg/kg TS
Aluminum	mg/L	NA	NA	NA	NA	NA	NA	NA	0.60	NA	NA

NA—not analyzed.

## 3. Membrane Technologies for Nutrient Recovery from LFD

Membrane technologies have become indispensable in the treatment and recovery of nutrients from LFD, mainly due to their selective separation capabilities and adaptability to different applications. These technologies include a range of processes driven by pressure, concentration gradients, vapor pressure or electric fields, each offering unique advantages in the recovery of nutrients. Pressure-driven processes, including MF, UF, NF and RO, are commonly used for the separation and concentration of nutrients in digestate. MF is often used to remove SS in LFD pretreatment and to reduce the pollution potential of downstream processes. By effectively separating the digestate into a solid-rich retentate and a nutrient-rich aqueous permeate, MF prepares the LFD for downstream membrane processes that concentrate valuable chemicals [2]. After the removal of SS and macromolecules, permeates are often rich in K and N and are, therefore, suitable as components of green fertilizers. The ability of RO and NF membranes, which enable the removal of smaller organic molecules and ions, to concentrate nutrients also allows for the more efficient recovery of resources, reducing waste while increasing the nutritional value of the retentate.

Processes driven by concentration gradients or vapor pressure, such as FO, pervaporation or MD, offer specific solutions for the concentration and recovery of nutrients. MD, a thermally driven process, has proven to be very effective in recovering nutrients and has little tendency to foul. MD is particularly valuable for the recovery of ammonia and the enrichment of P. For example, direct contact MD has achieved >99% removal of the total P from an AD effluent [26,27,28]. GPMs, designed to selectively pass gases (e.g., ammonia gas) while blocking liquids, are hydrophobic and less susceptible to fouling than conventional pressure-driven membranes. Due to their selectivity, GPMs are particularly effective for ammonia recovery, making them ideal for applications requiring high-purity ammonia extraction [29,30]. ED is an electrically driven membrane process that uses an electric field to selectively transport ions through membranes, making it ideal for concentrating nutrients from digestate. ED has proven successful in concentrating ammonium and other ions and allows the recovery of N- and K-rich streams suitable for fertilizer production. This process also facilitates the separation of specific ions from complex solutions and is, therefore, suitable for various nutrient recovery applications [31].

Membrane-based processes, particularly MF and UF, are increasingly being combined with biological treatment in membrane bioreactor (MBR) systems for digestate treatment to enable the removal of SS and the recovery of clean water. MBR systems provide high-quality effluents but tend to mineralize nutrients, which can limit nutrient recovery [3].

Overall, membrane technologies, including MF, UF, NF, RO, FO, MD and ED, offer versatile, effective solutions for the separation and concentration of valuable nutrients in the digestate and the simultaneous production of clean water. These processes convert the LFD into valuable resources for other industrial and agricultural applications and effluents suitable for further processing, contributing to a circular economy approach to nutrient management and recovery from anaerobic digestate [3].

### 3.1. Pressure-Driven Membrane Technologies

Pressure-driven membrane technologies are advantageous for the recovery of nutrients from anaerobic digestate [32,33], since they can recover 75–100% of the total ammoniacal nitrogen (TAN) [34] and 87–98% of P [35,36]. These physical separation processes are used to separate the LFD into two main streams: a solid fraction (retentate) and a nutrient-rich liquid fraction (permeate). These technologies include various membrane types, including MF, UF, NF and RO, each operating under increasing pressure and targeting increasingly smaller particles.

MF and UF membranes are generally used for the first stages of LFD separation. MF membranes (pore sizes 0.1–10 µm, pressure 0.1–3.0 bar) remove larger suspended solids, colloids and bacteria, while UF membranes (pore sizes 0.001–0.1 µm, pressure 2–10 bar) filter out dissolved organic compounds with a large molecular weight and colloidal particles [37]. In both processes, SS and macromolecules are removed with efficiency of over 80% [38]. UF allows for the achievement of TS-free permeates and 75% permeate recovery [2]. Dissolved compounds such as NH_4_^+^-N remain in the permeate [39]. The use of MF and UF reduces the risk of fouling in the downstream filtration membranes and improves the separation efficiency [1].

For the purification of MF and UF permeates, NF and RO membranes are usually used [3]. NF membranes, whose pores are smaller than 1 nm, retain small organic molecules and divalent ions and effectively concentrate nutrients such as P while allowing some ions such as ammonium to pass through. Studies report 5–23% NH_4_^+^-N retention and P retention of up to 97%, making NF very effective for P concentration as a result of the negative charge and large hydrated radii, which combine the effects of electrostatic repulsion and steric hindrance [40]. RO, with even smaller pore sizes, retains almost all dissolved solids and produces a highly purified water stream suitable for discharge or reuse. RO achieves nutrient retention rates of nearly 99–100%, effectively concentrating the N and P in the retentate and producing a permeate suitable for reuse [34]. However, the high pressure requirements (10–100 bar) and energy demands (approximately 4–6 kWh/m^3^) limit the scalability of RO, although it remains a valuable option for nutrient recovery and water reuse [41].

A combination of UF and RO allowed the removal of N and P of about 75–95% and 85–99%, respectively [1]. In the same type of system, the nutrient-rich retentate was suitable for producing fertilizer with content of 8.2–12.0 kg TN/t and 5.6–10.4 kg P_2_O_5_/t, whereas the permeate was rich in ammonium (2.9–5.6 kg NH_4_^+^-N/t) and K (6.2–9.2 kg K^+^/t) [37]. In the UF-RO system, the energy consumption reached 20–30 kWh/m^3^ digestate [37]. In another study, the integration of MF, UF and NF resulted in the recovery of 94.35% of N, and the final product served as a nutrient source for *Chlorella vulgaris* growth at the pilot scale [42].

To improve the efficiency of nutrient recovery, pressure-driven membrane technologies are often combined with additional treatment methods. For example, they can be combined with chemical precipitation to simultaneously recover N and P. Almost complete P recovery can be achieved by precipitating P through vivianite under optimum conditions (neutral pH and Fe/P molar ratio of 2.1). Next, N recovery with polyelectrolyte-modified NF membranes was also investigated: the ammonia selectivity was twice as high as that of a non-modified NF membrane [43]. Another example was a combination of RO with ammonia stripping [44].

Advanced configurations, such as vibratory shear-enhanced processing (VSEP), use RO membranes to remove macronutrients (N, P, K) from the LFD. VSEP RO filtration has demonstrated high efficiency in the removal of N (93%) and P (59%), although further optimization is required to meet regulatory discharge standards. The nutrient-rich retentate produced via VSEP can serve as a sustainable substitute for synthetic fertilizers and promote the circular management of nutrients [45].

Pressure-driven membrane technologies, including MF, UF, NF and RO, offer promising solutions for nutrient recovery from digestate. Each step is carefully tailored to concentrate and separate specific components. When combined with chemical or advanced filtration techniques, these processes optimize nutrient recovery for both agricultural reuse and environmental compliance, contributing to sustainable management practices in AD systems.

High-pressure systems, especially RO, consume large amounts of energy. Energy-efficient designs and the integration of renewable energy are essential in making these technologies more sustainable. The optimization of the membrane materials and operating parameters, such as the transmembrane pressure (TMP), cross-flow velocity or temperature, can reduce the energy costs and improve the separation performance [46].

Unlike RO, FO does not require high pressure for separation, as its driving force is the osmotic pressure difference between two solutions separated by a semipermeable membrane. FO membranes are less susceptible to fouling than other pressure membranes. However, the retention of total N by FO is low: although it is electrostatically attracted by the negatively charged surface of the membrane, only about 40% of NH_4_^+^-N is rejected because of its small radius [47]. However, it was possible to effectively reject P (>99%) and NH_4_^+^-N (>93%) with an FO membrane due to the formation of struvite [48].

### 3.2. ED

ED is a membrane-based electrochemical process that facilitates the concentration and separation of ions in liquids by applying an electrical field to move ions through ion-permeable membranes. In an ED system, anion exchange membranes (AEMs) and cation exchange membranes (CEMs) are placed alternately between the cathode (negative electrode) and the anode (positive electrode), creating compartments for dilute and concentrated solutions. In this configuration, the cations migrate through the CEMs to the cathode, while the anions migrate through the AEMs to the anode. This enables selective ion migration and concentration depending on the applied electric field [31].

ED processes can be operated at a constant voltage or constant current, which influences the energy efficiency and nutrient recovery rates. For example, during constant-voltage operation at 2.4 V/cell, removal efficiency of 92.8% was achieved, with NH_4_^+^ rejection efficiency of 43–65%, which was superior to the results obtained for other ions, such as K^+^ and Cl^−^. The optimal value was a constant cell voltage of 1.15 V, which allowed for about 93% total ion removal with energy usage of 0.44 kWh/m^3^. The energy consumption for N recovery ranged from 0.24 to 15.2 kWh/kg N, depending on the operating conditions, which affected ion migration and the corresponding removal efficiency [49].

Through ion migration, ED effectively concentrates ions such as NH_4_^+^, K^+^ and bicarbonate (HCO_3−_) in liquid digestate and produces an output solution with higher nutrient concentrations than RO [50]. Studies show that TAN concentrations of up to 16 g/L can be achieved with ED [51]. In combination with ammonia stripping, the yield can be increased even further, with concentrations of up to 21.356 g/L being achieved [52].

A hybrid process combining ED and electrochemical ammonia stripping (EAS) has been developed to improve ammonia recovery from anaerobic digestate. The ED-EAS system works by increasing the pH in the cathode compartment, which converts NH_4_^+^ into NH_3_, facilitating its recovery. In experiments with three ED cycles, ammonia was concentrated up to 3.775 g/L, three times the initial concentration, and a recovery rate of 90.5% was achieved with energy consumption of 11.6 kWh/kg NH_3_. Effective recovery was supported by optimal operating parameters, such as cathodic feeding and suitable current densities [53].

In addition to nutrient recovery, ED is also used for desalination and the production of organo-mineral fertilizers. For example, the treatment of ultrafiltered sugarcane vinasse with ED displayed high efficiency (>77%) in K recovery and enabled the production of K-based fertilizers, such as K-struvite, when combined with magnesium sulfate as an electrolyte solution. The addition of UF concentrate enabled the achievement of the required levels of K and organic carbon [31].

ED also facilitates the recovery of P from waste. The process has been used to dissolve and separate P from the organic fraction of municipal solid waste, achieving up to 43% P extraction [54]. The subsequent chemical precipitation of P in the form of struvite resulted in almost complete recovery, although heavy metals such as Zn imposed limitations for some agronomic applications.

ED has also been explored for the extraction of nutrients to support the production of single-cell proteins. In a hybrid electrochemical–membrane fermentation process, ED extracted 42.2% acetate and 60.1% ammonium from synthetic digestate, which was subsequently concentrated 14 and 10 times, respectively, by FO. This process provided nutrient streams for the cultivation of *Saccharomyces cerevisiae* and delivered amino acid levels above the FAO recommendations [55].

In a relevant study, micro- and ultrafiltrated LFDs from biogas plants processing agricultural waste, urban sewage sludge and animal manure were exposed to ED for the recovery of nutrients, which resulted in 51–67.8% recovery of N. To oxidize pharmaceuticals simultaneously, ozonation was used in the system. Applying this process before nutrient recovery was two times more efficient than applying it afterwards. This was due to the promotion of OH⋅ radical production from ozone by some metal ions present in the LFD [56]. In another study, which explored the use of a hybrid system explored for LFD from the treatment of pig manure, MF and UF were followed first by ED to recover ammonium (reaching 51%) and K, and then by RO to recover clean water [57].

The great potential of combined systems has been shown by the operation of another combined system using GPM technology for the recovery of N as ammonium sulfate and ED for P recovery. The N and P recovery from swine manure was 53 and 100%, respectively, and, for swine manure digestate, the respective values were 94 and 74% [58].

### 3.3. MCs

**Figure 1:**
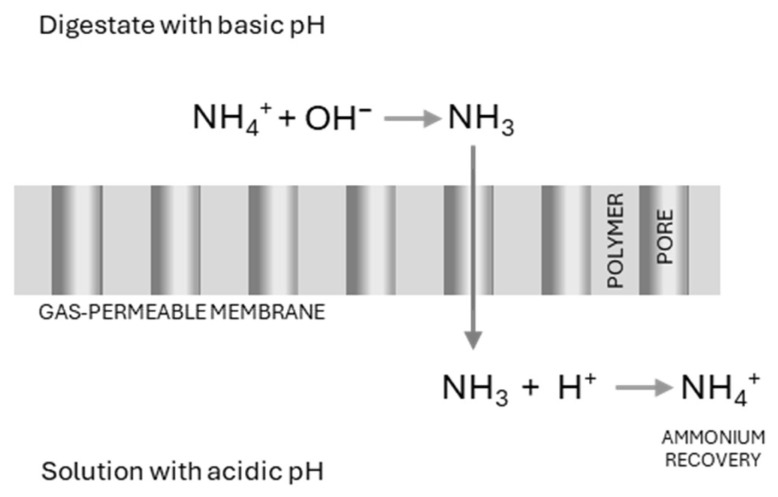
Principles of operation of GPM.

Depending on the pH and temperature, ammonia in LFD is present in two forms, volatile free ammonia (NH_3_) and ammonium ions (NH_4_^+^). Rising the pH promotes the conversion of NH_4_^+^ into NH_3_. In an MC system, effective ammonia recovery requires conditions that favor the volatile NH_3_ form, which can be achieved by increasing the pH and/or temperature of the wastewater [59]. Once the NH_3_ is in gaseous form, it diffuses through the hydrophobic gas-permeable membrane under low pressure (GPM, Figure 1), driven by the concentration gradient between the influent solution and the acidic permeate solution. The ammonia gas in the acidic solution, which is typically sulfuric acid (H_2_SO_4_), is then captured on the permeate side to form ammonium sulfate, a valuable nitrogen fertilizer [26,59].

This approach uses membranes that are usually composed of hydrophobic polymers such as polypropylene (PP), polyvinylidene fluoride (PVDF) or polytetrafluoroethylene (PTFE), which are highly permeable to NH_3_ [60]. MCs are usually configured in hollow fiber structures that have a high surface area to volume ratio, improving the mass transfer rates [61]. In these systems, the acidic solution circulates counter currently in the lumen of the fibers, which increases the contact time and promotes ammonia uptake. Studies show that a hollow fiber MC with the correct configurations can achieve ammonia removal efficiencies of 96–98%, as observed during the treatment of digestate and other ammonia-rich wastewaters [62,63].

Key factors influencing ammonia recovery include the pH, flow rate and membrane material. High pH values promote NH_3_ volatilization, while higher flow rates on the feed side improve the contact efficiency and diffusion rates. PTFE membranes with a nominal pore size of 0.22 µm in flat sheet configurations, for example, have shown efficient ammonia recovery (up to 71.6% over 3.5 h) with H_2_SO_4_ at a concentration of 1 M [64]. To achieve high efficiency in ammonia rejection, the specific membrane area should be as large as 32 m^2^/m^3^ [65].

The acid on the permeate side of the GPM can vary depending on the desired product. For example, using sulfuric acid produces ammonium sulfate, while using phosphoric or nitric acid produces ammonium phosphate or ammonium nitrate, both of which are used as commercial fertilizers. This flexibility makes MC adaptable to produce different ammonium-based fertilizers, depending on the agricultural needs [60].

Pilot-scale applications have confirmed the effectiveness of MCs in large-scale ammonia recovery. For example, a Danish pilot system combining a polypropylene hollow fiber MC with a UF pretreatment stage demonstrated ammonia removal efficiency of 85–90%, highlighting the feasibility of this technology for full-scale operation [44]. In the pilot-scale biogas plant, the recovery rate of TAN reached 16.2 g N/m^2^·d with recovery efficiency of 55.3%. The concentration of TAN in the trapping solution was 14-fold higher than in the LFD [66]. In another pilot study that used three MCs in series with sulfuric acid circulating in the lumen, the system achieved ammonia removal efficiency of 95% and produced fertilizer-grade ammonium sulfate as a by-product [67].

To improve nutrient recovery from LFD, various hybrid technologies have been developed. For example, the integration of ED and GPMs enabled 81% N recovery and 74% P recovery [68]. The integration of a GPM for N recovery and chemical precipitation for P recovery resulted in 77% N recovery and 80% P recovery (flux of 180 g N/m^2^ of GPM; addition of NaOH and MgCl_2_ as the precipitation agent) [69].

Innovative approaches, such as the integration of GPMs with air injection, have further optimized ammonia recovery in MC systems. By injecting air into the feed solution, additional reactions between air and bicarbonate in the fermentation substrate generate carbon dioxide and hydroxyl ions, which increase the pH and ammonia volatilization while reducing the need for chemical pH adjustment [70].

MC systems offer environmental benefits by reducing the ammonia emissions from wastewater, which can otherwise contribute to eutrophication and air pollution. In addition, by recovering ammonia in the form of fertilizer, these systems support circular nutrient management and reduce the need for synthetic, fossil fuel-derived fertilizers. Economically, MCs reduce the disposal costs for wastewater containing ammonia and produce valuable fertilizer products that provide an additional source of revenue for wastewater treatment plants and farms.

### 3.4. MD

MD is a thermally driven membrane technology that enables the separation and concentration of volatile components from wastewater streams. Using a microporous, hydrophobic membrane, MD utilizes the vapor pressure gradient created by heating the feed solution [71]. A heated feed solution flows along one side of a hydrophobic membrane, while a cooler permeate stream flows on the opposite side. This gradient drives the transport of water vapor and other volatile substances, such as ammonia, through the pores of the membrane, while non-volatile impurities and water remain in the liquid phase. This process results in a purified permeate on the distillate side, while nutrients such as phosphate remain concentrated in the feed solution, which improves nutrient recovery [72]. The attractiveness of MD in wastewater treatment lies in its high selectivity for ammonia, low-pressure operation and compatibility with low-grade or renewable energy sources [73].

Different MD configurations can be used: direct contact membrane distillation (DCMD), vacuum membrane distillation (VMD), air gap membrane distillation (AGMD) and sweep gas membrane distillation (SGMD).

In DCMD, the permeated component condenses directly in the liquid coolant. Both the feed and permeate solutions are in direct contact with the membrane, allowing for effective nutrient concentration in the digestate and the concentration of ammonia in a permeate solution while minimizing the permeate volume. DCMD is characterized by a simple configuration, easy operation and stable flux. DCMD is suitable for the treatment of food waste effluents, pharmaceutical wastewater, low-level radioactive wastewater, oily wastewater, wastewater containing suspended solids, organics, ammonia, P and pathogenic bacteria, brine solutions or produced water [74,75]. DCMD can be performed in a two-stage system, in which ammonia and water penetrate the membrane in the first stage to separate ammonia, whereas, in the second stage, ammonia is concentrated because only water penetrates the membrane. This approach has been used to concentrate ammonia from LFD, with significant ammonia flux achieved by operating at a higher pH and temperature [76]. With a temperature increase from 50 to 70 °C, the transmembrane flux was increased by three times [77].

In VMD, a vacuum is applied to the permeate side to increase the driving force for vapor transport, and the permeate is aspirated into the vacuum system. VMD has shown high efficiency in the removal of ammonia from digestate. For example, at a modest temperature of 45 °C and pressure below 20 mbar, more than 85% recovery of ammonia was obtained from LFD with a low TAN concentration of 200 mg/L [78]. VMD is usually operated under a larger TMP difference (up to 100 kPa) than any other MD configuration [79]. Because of these high pressures, the pore size is an important factor in separation [79], and the membranes used in VMD are more easily wetted [80]. Due to membrane wetting leading to reducing water flux, VMD is still under development [78].

AGMD incorporates an air gap between the membrane and the condensing surface on the permeate side, reducing thermal losses and improving the energy efficiency. The permeate passes through a layer of static gas and condenses on a cold plate, and the condensate is drained out of the module by gravity. AGMD has already been successfully used for nutrient concentration and water recovery from digestate. More than 98% of COD, P and K was removed, and TAN was almost completely removed [81]. The use of AGMD can increase the coefficient of NH_3_ transfer by seven times in comparison with conventional MD [82]. Although AGMD offers high efficiency in thermal energy utilization, it has low flux [74].

In SGMD, the water vapor is transported from the permeate side to an external condensation unit by sweep gas flow to maintain a TMP difference. SGMD combines minimized conductive heat loss with minimal mass transfer resistance. The type of membrane affects the SGMD performance due to the change in the sweep gas flow rate; it is particularly visible when using hollow fiber membranes, where the axial pressure gradient along the membrane is more significant than for flat sheet membranes [72].

Because ammonia is a highly corrosive chemical, the membrane lifespan is of particular importance. The presence of ammonia may reduce the hydrophobicity of the membrane. It was found, however, that there was no static contact between ammonia and the membrane in SGMD, and the fast removal of ammonia by sweep gas flow minimized membrane damage [72].

For the purpose of ammonia removal, VMD and SGMD are apparently promising, since the volatile substances can be collected in a condensation unit without the aid of extra chemicals. DCMD is less competitive, because an additional acid permeate solution is needed to collect the permeated ammonia, increasing the chemical consumption [80].

When comparing three MD configurations, the following order was identified based on the mass transfer coefficient: VMD > DCMD > SGMD. Moreover, based on the selectivity, this was DCMD > SGMD > VMD. The highest mass transfer coefficient for VMD makes this configuration the optimal one for the kinetics for ammonia mass transfer. The VMD conditions promote also thermodynamics for water evaporation, thus resulting in much higher water flux and smaller conductive heat losses than in DCMD and SGMD [78,79]. Due to the vacuum, VMD operates at lower temperatures, resulting in the reduced consumption of energy, which is lower than for DCMD and AGMD [83].

VMD, DCMD and SGMD have been extensively used for stripping purposes. It was also found that DCMD is characterized by the relatively higher thermal capacity of the liquid on the permeate side, which makes it less sensitive to the feed temperature [79]. For all three MD configurations, higher mass transfer coefficient values but lower selectivity are observed in the feed at higher temperatures. In all three MD configurations, lower selectivity can be found in feed with a higher ammonia concentration [79].

A comparison of the different configurations of MD in terms of the ammonia recovery rate, energy consumption and thermal efficiency (ratio of heat of vaporization to total heat) is presented in Table 2.

Key factors affecting ammonia recovery in MD include the feed solution pH, the temperature and the ammonia vapor–liquid balance. Increasing the pH of the feed solution shifts the ammonia balance towards NH_3_ gas, which increases its volatility and allows higher transport rates through the membrane. In addition, higher feed temperatures increase the vapor pressure gradient, which increases the flow rate and improves the efficiency of ammonia recovery. For example, a DCMD system operated at a pH of 12 and a temperature of 60 °C achieved ammonia removal efficiency of 84.2%, with the ammonia concentration in the gas reaching up to 26.3 g/L after several batches [76].

MD systems require thermal energy to heat the feed and to cool the permeate and electrical energy to drive the pumps. To industrialize this process, the thermal efficiency should be improved to reduce the energy consumption. The low temperature requirements of MD make it compatible with low-quality heat sources, such as solar energy or residual heat from AD systems. By coupling MD with AD, the waste heat from biogas combustion can power the MD process, providing a sustainable solution for nutrient recovery. This integrated system has been applied to the treatment of livestock wastewater and has shown strong potential in recovering nutrients while efficiently utilizing the energy generated in the AD process [3]. Another method to efficiently utilize this energy is the use of MD to concentrate the nutrient-rich stream as a second step after the hydrothermal liquefaction (HTL) of organic waste [93]. The residual heat from the HTL drove the MD process, resulting in very high concentrations of ammonium and P, as well as a reduced MD cost. The results show that, when MD utilizes a waste heat source, thermal efficiency is less of a concern [81]. Therefore, the integration of MD systems with sewage treatment systems, biogas plants that process organic waste or solar systems offers advantages such as lower energy consumption.

To increase the mechanical strength of the MD membrane and the ammonia recovery rate, modifications of the membranes have been investigated. For example, a PVDF membrane was modified by the incorporation of a copolymer of tetrafluoroethylene and perfluorosulfonic acid and multiwall carbon nanotubes [94]. As a result, the efficiency of ammonia recovery was three times higher than with a non-modified PVDF membrane.

To overcome limitations such as environmental pollution and energy requirements, MD is often combined with other processes. For example, MD-FO hybrid systems or systems with anaerobic bioreactors (MDBRs) have been developed to improve the nutrient recovery while reducing the environmental pollution. In particular, an MD-FO system can enable the simultaneous recovery of N, P and K, making it a valuable tool for comprehensive nutrient recycling in wastewater treatment [95].

### 3.5. Summary of the Use of Membrane Technologies for Nutrient Recovery from LFD

The selection of membrane technology to recover nutrients from anaerobic digestate requires the careful consideration of the recovery efficiency, energy consumption and the targeted nutrients (Table 3). Each membrane technology offers unique advantages and limitations, with differences in their performance, energy requirements and suitability for integration into hybrid systems. MF and UF are cost-effective pretreatment methods that achieve moderate nutrient recovery while preparing the digestate for downstream processes. MF recovers around 20% of N (NH_4_^+^) and P (PO_4_^3−^) with low energy consumption of 1.77 kWh/m^3^. UF has slightly higher recovery rates, especially for P (~30% NH_4_^+^ and ~60% PO_4_^3−^), but the energy consumption increases to 2.3–8.8 kWh/m^3^. These technologies are characterized by reduced fouling and ensuring the smoother operation of the downstream membrane processes.

Advanced membrane systems such as NF and RO offer significantly higher recovery efficiency, especially for P. NF can retain up to 97% of P using its ability to retain divalent ions, while the N recovery rates are modest (5–23%). Conversely, RO offers the almost complete recovery of N and P (99–100%) as it retains all dissolved solids. However, these high recovery rates come at the cost of increased energy consumption, which is between 4.3 and 30 kWh/m^3^ for RO. The significant energy requirements of RO and NF limit their standalone application but make them valuable components in hybrid configurations, where they can efficiently concentrate nutrients from pretreated streams, reducing the overall process costs.

ED and MD offer additional flexibility by targeting specific ions or utilizing thermal energy sources. ED is particularly effective in N recovery, achieving rates of 43–65% for NH_4_^+^ with relatively low energy consumption (0.44 kWh/m^3^). Integration with ammonia stripping or chemical precipitation further increases the recovery potential. MD, on the other hand, uses thermal gradients to achieve the almost complete recovery of N (up to 100%) and P (>99%), with energy consumption between 0.25 and 9.1 kWh/m^3^. This technology is particularly suitable for systems using low-grade waste heat or renewable energy and offers a sustainable route to nutrient recovery and water reuse.

The relationship between energy and nutrient recovery underlines the importance of tailoring membrane processes to specific operational objectives. Technologies such as MC and MD are ideal for maximizing nutrient recovery while minimizing energy consumption, especially in applications requiring high purity for fertilizer production. Conversely, MF and UF offer efficient pretreatment options with minimal energy requirements, making them indispensable in hybrid systems. The adaptability of these technologies ensures their compatibility with different raw materials and operating conditions, which promotes their adoption in circular economy strategies.

**Figure 2:**
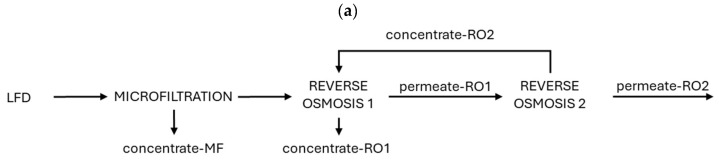

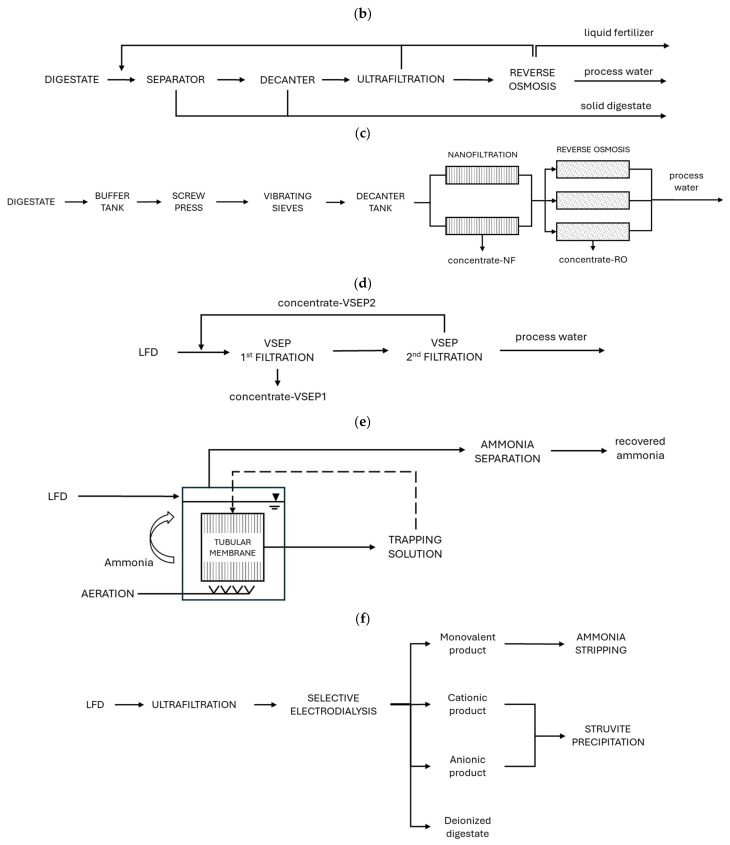

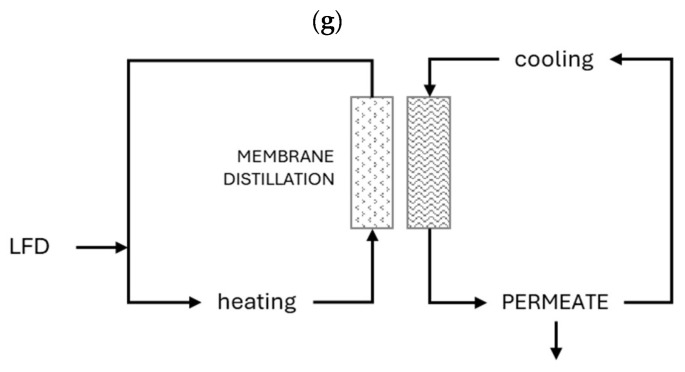
Membrane technologies: (**a**) MF-RO, (**b**) UF-RO, (**c**) NF-RO, (**d**) VSEP, (**e**) MC, (**f**) ED, (**g**) MD.

Figure 2 presents some examples of the introduction of membrane technologies into the LFD treatment process.

## 4. Fouling in Membrane-Based Processes

Fouling is an important limiting factor in the application of membrane technologies for nutrient recovery from LFD. It consists of the deposition of organic, inorganic and microbial materials on the membrane surface or in its pores, which significantly reduces the efficiency and increases the operating costs. This phenomenon poses a challenge in maintaining long-term performance and limits the scalability of these technologies. Despite promising results in laboratory studies, fouling remains a major obstacle to full-scale implementation [105].

Membrane fouling can be categorized into four main types: organic, inorganic (scaling), biofouling and particulate fouling. Organic fouling is caused by the accumulation of extracellular polymeric substances, soluble microbial products and other organic compounds such as humic acids and proteins. These substances form biofilms or cake layers that reduce the permeability of the membrane. Inorganic fouling is caused by the precipitation of poorly soluble salts such as calcium and magnesium phosphates or silicates, which form hard deposits on the membrane surface. Scaling occurs primarily at high pH values and temperatures. Biofouling is the result of the microbial colonization of membrane surfaces, which leads to the formation of a biofilm that impedes mass transfer and fluid flow. Elevated levels of ammonium and phosphate in the LFD can accelerate this process [106]. In addition, certain membrane materials, such as polyamide-based RO membranes, can adsorb proteins and microbial debris, which further promotes biofilm maturation [107]. Particulate fouling is caused by suspended solids or colloidal substances that clog the pores of the membrane [73]. Colloids in the 0.3–1 µm range, including residual lignocellulose fragments from the digestate, can block membrane pores, and those within the range of 10–1000 μm contribute to the formation of a cake layer [108].

Detailed investigations of fouling, such as molecular-size-specific analysis, provide insights into the predominant foulants and help to develop targeted mitigation strategies. In ED membranes, AEMs were mainly fouled by dissolved organic matter with a molecular size smaller than 10 kDa; in particular, particles smaller than 1 kDa penetrated the interior of the membrane and increased the membrane resistance by 25.7% [109]. Particles larger than 10 kDa did not cause fouling. CEMs were not fouled, irrespective of the size of the organic particles. According to Yan et al. [110], the deposition of magnesium-, calcium-, phosphate- and silicon-related compounds was the main reason for membrane scaling in MD, while organic fouling was mainly attributed to the adsorption of organic compounds on inorganic scaling. In contrast, fouling in pressure-driven membranes is caused by high-molecular-weight organic particles; the fouling of UF and RO resulted from particles of about 100 kDa [111].

Fouling significantly influences membranes’ performance, including reduced permeability, increased TMP and higher energy consumption. Foulant accumulation necessitates the frequent cleaning or replacement of membranes, which increases the operating costs. UF systems for the treatment of digestate, for example, can incur maintenance costs of EUR 4 to EUR 12/m^3^ due to fouling [1]. Overcoming fouling challenges requires a multi-faceted approach, including the further development of membrane materials, optimization of pretreatment processes and integration of hybrid systems.

Various strategies have been developed to reduce the fouling of membranes and extend their lives. The pretreatment of the feed is one of the most effective approaches. It includes coagulation/flocculation and advanced oxidation or enzymatic hydrolysis to remove organic and particulate matter [31,73]. Fe(III)/peroxymonosulfate pretreatment, for example, significantly reduces dissolved organic carbon and minimizes fouling in MD processes [112]. Ozone pretreatment reduces the biopolymer concentration and the viscosity of the digestate and increases the permeability of UF systems by 50–80% [113]. To diminish fouling without using chemicals, the hydrothermal treatment of LFD was developed; a nine-hour process at temperatures of 120 and 220 °C affected the viscosity of the LFD and improved the filterability [114]. The integration of thermal hydrolysis in AnMBRs has shown that it is possible to reduce the sludge viscosity and fouling while maintaining a stable flow rate and low TMP [115]. Enzymatic treatments with amylase, pectinase, cellulase and protease improve the filterability of fermentation substrates and reduce the risk of fouling in the subsequent filtration stages [37]. These pretreatments not only improve the filtration efficiency but also reduce the energy and maintenance requirements of membrane systems. In addition, diminishing inorganic scaling by the acidification of the feed resulted in decreased organic fouling [110].

Continuous innovation in self-cleaning technologies is crucial in improving the feasibility and sustainability of membrane-based processes for wastewater treatment. Cleaning protocols, including chemical agents such as citric acid and sodium hypochlorite, help to restore the membrane flux by 20.65 and 11.95%, respectively, and eliminate accumulated foulants [116]. Regular physical cleaning, such as backwashing and ultrasonic treatments, is also used to remove particulate fouling. In DCMD, for example, fouling by organic and inorganic substances requires frequent cleaning cycles with acidic and alkaline agents, which further increases the operating costs [116]. Self-cleaning mechanisms such as electrodialysis reversal utilize polarity switching to reduce fouling on ion exchange membranes [109]. In addition, operational adjustments such as optimizing the cross-flow velocities can reduce the risk of fouling, although excessive velocities can increase the energy consumption [113]. Non-contact MD, where an air gap is introduced between the membrane and the feed solution, also offers a promising solution to minimize fouling [82].

The development of antifouling membranes has become an important focus in addressing the challenges associated with the treatment of the liquid fraction of digestate. Among these innovative approaches, FeOOH-grafted nanomembranes have proven to be a significant advancement [117]. In these membranes, FeOOH nanoparticles are grafted onto polyvinylidene fluoride (PVDF) substrates, forming a reentrant nanostructure that improves both the antifouling and antiwetting properties. The grafting process, which involves hydrothermal synthesis and modification with fluorosilane, increases the hydrophobicity of the membrane, achieving a contact angle of 152.9°, compared to 112.8° for unmodified membranes. This increased hydrophobicity effectively minimizes pore wetting and prevents scaling and organic fouling, which are major challenges in digestate treatment [117].

In DCMD tests, FeOOH-grafted membranes showed remarkable performance. They achieved ammonia recovery rates of 95.2% and ammonia flux of 3.89 g/m^2^·h, clearly outperforming the unmodified PVDF membranes (2.45 g/m^2^·h). In addition, these membranes maintained their structural integrity and operating efficiency over extended periods of time, with minimal fouling and the negligible leaching of FeOOH nanoparticles (0.4% on the feed side). This stability is critical in addressing the complex composition of digestate, which is rich in proteins, human acids and inorganic salts [117].

In addition to this innovation, membranes modified with carbon nanotubes (CNTs) offer another advanced solution for the treatment of liquid digestate. CNT membranes are known for their high mechanical strength, thermal stability and exceptional antifouling properties and show improved performance in treating the high concentrations of ammonia, salts and organic matter in digestate. Functionalized CNTs, such as carboxylated CNTs (f-CNTs), improve the hydrophilicity and ammonia sorption. Studies show that CNT-modified membranes achieve up to 63% higher ammonia flux compared to conventional PTFE membranes, while exhibiting higher efficiency in the removal of ammonia and salts. These properties are crucial for the recovery of nutrients and the reuse of water in waste management [90].

In addition, CNT modifications create superhydrophobic surfaces that significantly reduce biofouling and organic deposits. The contact angle of f-CNT membranes in aqueous ammonia solutions decreases to 80°, indicating improved hydrophilicity that minimizes foulant adhesion. Importantly, these membranes exhibit robust flux recovery and retain up to 80% of their performance after purification, compared to 60% for unmodified membranes [118]. Although challenges such as the cost and uniform CNT dispersion remain, the high rejection rates, longevity and ability to handle complex wastewater compositions highlight their potential for scalable digestate treatment [119].

Another promising approach is sulfonated graphene nanofiltration membranes. These membranes, functionalized with sulfonic acid groups, exhibit excellent hydrophilicity, which increases the water flux and reduces fouling by organic matter and microbial cells. The strong electrostatic repulsion that occurs between the negatively charged sulfonated surfaces and the fouling substances further minimizes clogging. Improved salt retention and contaminant removal allow for the efficient recovery of clean water and valuable nutrients. Optimized configurations of these membranes have shown improvements in water flow of up to 11.8 L/m^2^·h, representing a significant increase in efficiency over conventional systems [120].

Polydopamine (PDA)-coated membranes are also an effective approach to overcoming the challenges of fouling, wetting and mechanical instability in digestate processing. PDA coatings produced by the self-polymerization of dopamine significantly improve the hydrophilicity of the membranes by reducing the contact angle by 20–30% and forming a hydrated surface layer that prevents the adhesion of organic and microbial material. These coatings also improve the mechanical strength by up to 300%, ensuring a long service life under harsh operating conditions. In addition, PDA provides a versatile platform for further functionalization and improves the antifouling and nutrient recovery capabilities. Thanks to their scalability and cost-effectiveness, PDA-coated membranes are ideal for the sustainable liquid fraction of digestate [121].

Finally, the grafting of zwitterionic polymers represents an innovative strategy for the development of antifouling membranes. By attaching polymers such as sulfobetaine methacrylate, these membranes obtain highly hydrophilic and charge-neutral surfaces that form a hydration layer that prevents organic fouling and the adhesion of proteins and microbial cells. This modification reduces fouling by up to 90%, resists salt deposits and improves the water flow and solute removal. The longevity of zwitterionic membranes under various conditions makes them particularly effective in overcoming the challenges of digestate treatment, including fouling and scaling, while improving the nutrient recovery and water reuse efficiency [122].

The integration of metal oxide nanoparticles such as TiO_2_, ZnO and Al_2_O_3_ into membrane systems has proven to be a promising strategy to reduce fouling in the treatment of liquid digestate. TiO_2_ nanoparticles exhibit photocatalytic activity as they degrade organic contaminants, while ZnO has an antimicrobial effect and reduces biofilm formation by 65% [123], and Al_2_O_3_ increases water flow by 30% by disrupting the packing of polymer chains [124]. These nanoparticles improve the hydrophilicity of the membranes, reduce the contact angle by 10° and reduce the fouling resistance by up to 40% [125]. For example, ZnO-coated multiwalled carbon nanotubes in polyethersulfone membranes increased the water flux by 25% and reduced the biofilm thickness by 50%, while TiO_2_ nanoparticles improved the solute retention by 20% and minimized the flux decline by 20% [126].

Hybrid systems combining FO and MD are a promising solution for the treatment of the liquid fraction of digestate, as they offer efficient water recovery, nutrient concentration and reduced fouling [95]. In the hybrid system, FO is used to concentrate the liquid fraction of the digestate by drawing water at high osmotic pressure through a semipermeable membrane into a draw-off solution (DS), leaving a concentrated feed solution enriched with nutrients. MD then uses low thermal energy to recover the water by separating the vapor from the diluted DS while regenerating the DS for reuse in the FO process. This cyclic integration ensures consistent performance, high water recovery and minimal fouling compared to standalone systems. Studies have shown that FO-MD systems can achieve water recovery rates of more than 80%, with the high retention of salts, ammonia and organic impurities, making them particularly suitable for the complex composition of the liquid fraction of digestate [127].

The FO-MD hybrid system has robust nutrient concentration capabilities, which are essential for downstream processes such as struvite precipitation or ammonia recovery. For example, FO membranes concentrate the digestate and significantly increase the ammonia content for efficient recovery, while MD membranes prevent DS contamination and scaling by selectively allowing the permeation of water vapor. This approach addresses the high fouling tendency of digestate as FO is inherently resistant to fouling as it operates without hydraulic pressure and forms a hydration layer that minimizes the deposition of solutes on the membrane surface [128].

In a practical application, FO-MD hybrid systems were successfully used in the treatment of dairy wastewater treatment, which shares compositional similarities with digestate. These systems achieved flux recovery of over 90% after membrane cleaning and showed consistent performance despite the high organic load, proving their applicability in the treatment of digestate [127]. The use of sodium chloride as the target solution enabled effective water flux and DS regeneration, while MD ensured high-quality water recovery without significant energy inputs [128].

Although FO-MD systems offer numerous advantages, their application in the treatment of the liquid fraction of digestate faces challenges, such as addressing the strong variability in the feedwater composition and optimizing the DS regeneration performance. Advanced DS compositions, such as thermolytic solvents or fertilizers, could further improve the cost efficiency and susceptibility of the system [129]. In addition, the integration of FO-MD with other nutrient recovery technologies, such as electrodialysis or chemical precipitation, could improve the overall resource recovery and reduce waste generation.

Despite these advances, fouling is still a bottleneck in the expansion of membrane technologies. Although pressure-driven membrane technologies are beneficial, the high costs associated with cleaning, pretreatment and energy consumption continue to hinder their widespread application.

## 5. Biological Removal of N and Its Combination with Membrane Techniques

Biological processes for N removal are key technologies for N management in anaerobic digestate. These processes primarily include nitrification–denitrification and anaerobic ammonium oxidation (anammox), each of which has different mechanisms and environmental impacts. Despite their effectiveness, challenges such as greenhouse gas emissions, limited N recovery and operational complexity remain, requiring continuous innovation and optimization.

The conventional nitrification–denitrification process involves two successive stages. In the first stage, ammonia–nitrogen (NH_4_^+^-N) is oxidized to nitrate (NO_3_^−^-N) by nitrification with the help of nitrifying bacteria. This is followed by denitrification, in which NO_3_^−^-N is reduced to gaseous nitrogen (N_2_) by denitrifying bacteria under anoxic conditions. While this method is effective in removing N, it requires significant energy input for aeration and often an external carbon source to maintain denitrification. In addition, this process releases nitrous oxide (N_2_O), a greenhouse gas with global warming potential that is 298 times higher than that of CO_2_, making its impact on the environment a critical issue [130].

The anammox process offers several advantages over conventional methods. It eliminates the need for an external carbon source and reduces the energy required for aeration by converting ammonia (NH_4_^+^) and nitrite (NO_2_-) directly into N_2_ in a single anaerobic step. This process also works in smaller reactor volumes, which improves the space efficiency. However, anammox does not allow ammonia recovery as it is ultimately removed as an inert gas instead of being captured for reuse [131].

Both nitrification–denitrification and anammox are effective but have their own limitations. The environmental impact of N_2_O emissions during denitrification undermines the sustainability of these processes. In addition, neither method allows the recovery of N in a reusable form, which is becoming increasingly important in the circular economy. Therefore, there is growing interest in integrating biological processes with physicochemical methods such as ammonia stripping, membrane separation and struvite precipitation to enable the recovery of N and minimize the impact on the environment [132].

**Figure 3:**
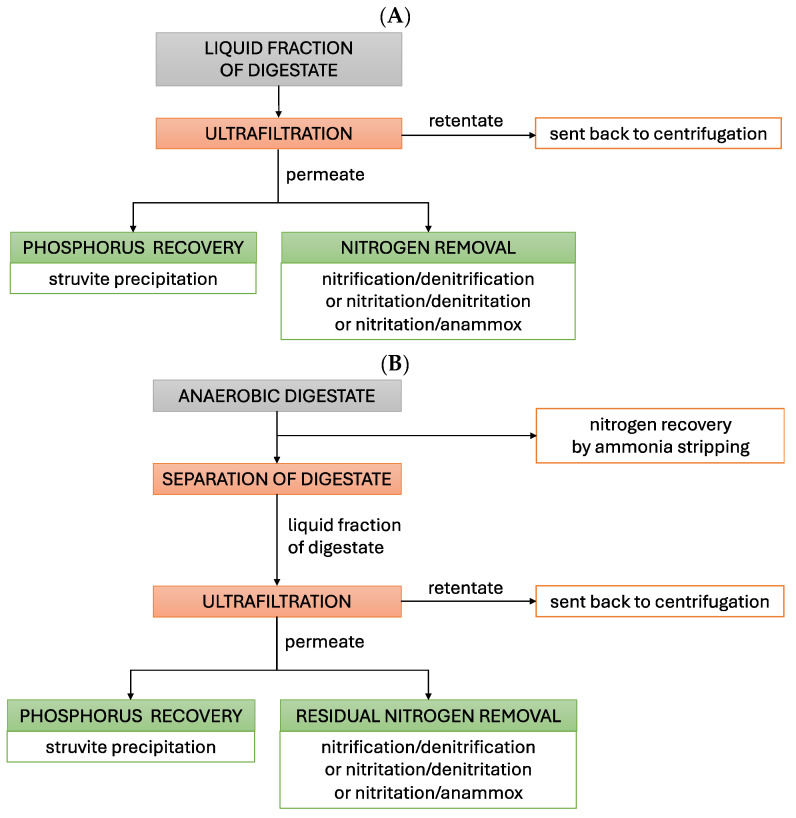
Incorporation of biological N conversion in P recovery (**A**) and N and P recovery (**B**).

Although the biological conversion of N limits N recovery as it usually ends with the release of N_2_ into the atmosphere, its introduction into the systems used for the membrane processing of LFD may support N and P removal/recovery [133]. In the variant presented in Figure 3A, LFD, which is obtained by, e.g., centrifugation, is subjected to UF. The permeate is exposed to struvite precipitation for P recovery, whereas biological processes are used to remove N from the final effluent. In the variant presented in Figure 3B, N is recovered from the digestate using ammonia stripping. The residual digestate is separated by, e.g., centrifugation. The obtained LFD is subjected to UF. The permeate is exposed to struvite precipitation for P recovery, whereas residual N is removed with the employment of biological processes.

Recent advances in the field of biological N removal have led to the introduction of the Ultrasonic System for Anaerobic Fluidized Bed Membrane Bioreactors (US-AnFMBR), which integrates state-of-the-art techniques to address the challenges of contaminant removal and operational stability. This novel system combines ultrasonic treatment with the addition of biochar to achieve significant improvements in N and P recovery while reducing membrane fouling [133]. One of the most notable achievements of the US-AnFMBR system is its improved range efficiency. The system successfully achieved removal rates of 89.41% for COD, 49.29% for NH_4_^+^-N and 54.83% for phosphate. These results highlight the system’s ability to effectively remove both organic and nutrient-related contaminants, making it a promising solution for wastewater treatment. Because the US-AnFMBR system targets multiple pollutants simultaneously, it provides a holistic approach to pollution control that is critical to maintaining ecological balance and mitigating eutrophication risks. A key feature of this system is the integration of biochar, a cost-effective and sustainable material derived from agricultural waste. At an optimal dose of 2.5 g/L, biochar improves the performance of the sewage sludge by adsorbing and degrading organic material. This not only improves the N removal from the system but also provides an innovative way to recycle agricultural waste. The dual functionality of biochar—the removal of pollutants and the recycling of waste—is in line with the principles of the circular economy and demonstrates its potential for further environmental applications. The use of ultrasound further differentiates the US-AnFMBR system from other systems. By applying ultrasonic energy (30 min at 26 W), the system activates the microorganisms in the sludge and improves their ability to dissolve and degrade pollutants. This process reduces the sludge’s viscosity and facilitates the preferential adsorption of NH_4_^+^-N over PO_4_^3−^-P. The improved activity of the sludge microorganisms not only increases the efficiency of nutrient removal but also creates favorable conditions for subsequent membrane filtration, which reduces operational challenges. Another important advancement of the US-AnFMBR system is its ability to reduce membrane fouling, a common limitation of membrane-based technologies. The reduced viscosity of the sludge, combined with the improved solubility of contaminants, minimizes the accumulation of contaminants on the membrane surface. This improvement allows substances with small molecules to pass through the membrane more easily, ensuring stable operation over longer periods of time. The system’s ability to solve fouling problems increases its practicality for real-world applications where fouling is a significant barrier to efficiency and cost-effectiveness.

## 6. Pilot- and Full-Scale Applications of Membrane Technologies for Nutrient Recovery

Although membrane technologies have reached a technology readiness level (TRL) of 9, their full-scale implementation for nutrient recovery from digestate is still limited. Most full-scale applications focus on combining multiple membrane processes, often in conjunction with pretreatment steps, to achieve the efficient separation and recovery of N and P. Although promising results have been achieved at both a pilot and full scale, problems such as the operating costs and fouling continue to hinder widespread application.

In Italy, Bolzonella et al. [134] evaluated membrane-based nutrient recovery from digestate at two full-scale AD plants treating cow and pig manure and energy crops. The treatment system included screw press separation, UF and RO. The system achieved a recovery rate of over 50% for N and P, with 50% of the digestate mass being converted to clean water. For cow manure digestate, the RO concentrate contained 4.8 g N/kg and 0.36 g P/kg, while, for pig manure digestate, these concentrations were 5.27 g N/kg and 0.26 g P/kg, respectively. These results show that it is possible to produce concentrated fertilizers and clean water by sequential membrane separation.

In the Netherlands, Van Puffelen et al. [135] implemented a cascaded membrane filtration system at a full-scale facility processing agricultural digestate. The system included decanter centrifuges, MF, RO and an ion exchanger. This system achieved 98% P removal prior to RO, with 66% of the P recovered in the first solid fraction, which accounted for only 15% of the total mass. Further P recovery occurred in the MF concentrate, which was reused as a liquid organic fertilizer. N recovery was similarly effective: 34% of the TN was recovered in the RO concentrate. The implementation of this system reduced the transportation distance of the processed digestate by 53% compared to the raw digestate, demonstrating both economic and logistical benefits.

Pilot-scale studies have further validated the potential of membrane technologies for nutrient recovery. In Germany, Gienau et al. [136] tested a pilot plant with ceramic UF and a three-stage RO system. This system achieved almost complete nutrient recovery and produced high concentrations of dissolved ammonia (4 kg NH_4_-N/t) and K (10 kg K_2_O/t) in the RO retentate. The process also recovered 38% of the digestate volume as clean water and produced different fertilizer products: a solid N/P fertilizer and a liquid N/K fertilizer.

In Poland, Zielińska and Mikucka [36] reported on a pilot study that demonstrated the effectiveness of UF as the final step in nutrient recovery. The system achieved N and P rejection rates of 81% and 87%, respectively, highlighting the ability of UF to produce nutrient-rich concentrates.

## 7. Challenges and Costs of Membrane Technologies for Nutrient Recovery

Pilot- and full-scale studies show the technical feasibility of membrane technologies for nutrient recovery from digestate, which have significant potential to produce high-quality fertilizer products and clean water. However, challenges such as fouling, energy consumption and the operating costs need to be addressed to enable wider application. Continued innovation in pretreatment methods, energy recovery and hybrid system designs will be critical to scale up these technologies and ensure their economic and environmental sustainability.

Despite the promising results, there are still considerable obstacles to the widespread acceptance of membrane technologies. The operating costs for full-scale plants range between EUR 4 and EUR 12/m^3^ of the fermentation substrate, mainly due to energy consumption and maintenance costs [1]. For example, UF is the most energy-intensive step and consumes 10–15 of 20–30 kWh/m^3^ of the fermentation substrate. These costs necessitate the development of energy-efficient processes such as enzymatic pretreatment and ozone treatment to reduce the viscosity and improve the membrane performance [113].

In addition, fouling remains a major challenge in both pressure-driven and non-pressure-driven systems. Advanced pretreatment methods such as flocculation and enzymatic hydrolysis are crucial to reduce fouling and ensure long-term membrane performance.

While pressure-driven systems such as RO, NF, UF and MF are well established, non-pressure-driven membrane technologies are still in the early stages of development. These emerging methods, such as FO and MD, are promising for nutrient recovery but require further research and pilot-scale testing to be commercialized [1].

## 8. Conclusions and Future Perspectives on Membrane-Based Technologies

The recovery of nutrients from digestate is in line with the principles of the circular economy by converting waste streams into valuable products such as fertilizer and water. Membrane technologies have the potential for efficient nutrient recovery but face challenges such as energy requirements, membrane fouling and limited scalability. Overcoming these challenges is crucial for their wider application.

Key advances need to focus on optimizing the energy consumption, improving the membrane materials and developing hybrid systems to integrate nutrient recovery into broader wastewater management strategies. Innovations such as energy-saving and renewable energy-powered systems, combined with improved fouling-resistant membranes, can significantly improve the cost efficiency. Hybrid configurations that combine membranes with advanced biological and chemical processes promise to maximize the resource recovery efficiency while minimizing the environmental impact.

Economic feasibility remains a critical hurdle as the operating costs are high and market incentives for bio-based fertilizers are insufficient. Comprehensive techno-economic assessments are essential to validate the scalability, operational safety and cost efficiency under real conditions. The impact of nutrient application on the environment must also be considered, with a focus on sustainable agricultural practices.

Future research should focus on adaptable designs for different feedstock compositions, pilot-scale validations and integration with renewable energy systems. Exploring synergies with new technologies such as electrodialysis and advanced oxidation can further improve the system’s performance and the resource recovery opportunities.

## Figures and Tables

**Table 2:** Comparison of configurations of MD.

	Ammonia Recovery Rate (%)	Energy Consumption (kWh/m^3^)	Thermal Efficiency (%)
DCMD	98.72% [84]	1500 kWh/m^3^ [85] 436 kWh/m^3^ with a heat recovery system [86]	59.6–70.5 [87]
VMD	100% [88] <70% [89]	88 kWh/m^3^ (multi-stage system) [90]	88.1–91.9 [87]
AGMD	up to 100% [81]	900–1300 kWh/m^3^ without heat recovery, 66–170 kWh/m^3^ with heat recovery [81]	70.0–98.0 [87]
SGMD	85% [72]	1.09 kWh/kg [91]	~92.0 [92]

**Table 3:** Comparison of the most important performance indicators for different membrane technologies.

	Nutrient Recovery Rate	Energy Consumption
MF	~20% (NH_4_^+^, PO_4_^3−^) [38]	1.77 kWh/t [96]
UF	~30% (NH_4_^+^), ~60% (PO_4_^3−^) [38]	10–15 kWh/m^3^ [37] 2.3–8.8 kWh/m^3^ [97,98]
NF	5–23% (NH_4_^+^-N), 97% (P) [40] 94.35% (N) (MF–UF–NF) [42]	2.2 kWh/m^3^ [99] 4.5–11 kWh/m^3^ (UF-NF) [100]
RO	99–100% (N and P) [34] 75–95% (N), 85–99% (P) (UF–RO) [1] >85% (NH_4_^+^, PO_4_^3−^) [38]	4.3–5.5 kWh/m^3^ [101,102] 20–30 kWh/m^3^ (UF-RO) [37] 16–25 kWh/m^3^ (UF-RO) [103] 6.6–14.4 kWh/m^3^ (UF-RO) [100]
ED	43–65% (NH_4_^+^) [49] 90.5% (NH_4_^+^) (ED–electrochemical ammonia stripping) [53] 60.1% (NH_4_^+^-N) [55] 43% (P) [54] 51–67.8% (N) (MF–UF–ED) [56] 51% (NH_4_^+^-N) (MF–UF–ED) [57] 94% (N), 74% (P) (MC–ED) [58] 81% (N), 74% (P) (MC–ED) [68]	0.44 kWh/m^3^ [49] 11.6 kWh/kg NH_3_ [53]
MC	96–98% (NH_4_^+^) [62,63] 71.6% (NH_4_^+^) [64] 95% (NH_4_^+^) [67] 55.3% (TAN) [66] 85–90% (NH_4_^+^) (UF–MC) [44] 77% (N), 80% (P) (MC–chemical precipitation) [69]	0.049 kWh/m^3^ [104]
MD	>99% (P) [27] 100% (TAN), >98% (P) [81] 84.2% (NH_4_^+^) [76]	0.25 kWh/m^3^ [65] 2.5–9.1 kWh/m^3^ (UF-MD) [100]

## Data Availability

The original contributions presented in the study are included in the article; further inquiries can be directed to the corresponding author.
